# Quantitative Modeling of Spasticity for Clinical Assessment, Treatment and Rehabilitation

**DOI:** 10.3390/s20185046

**Published:** 2020-09-05

**Authors:** Yesung Cha, Arash Arami

**Affiliations:** 1 Neuromechanics and Assistive Robotics Laboratory, University of Waterloo, 200 University Ave W, Waterloo, ON N2L 3G1, Canada; y2cha@uwaterloo.ca; 2 Toronto Rehabilitation Institute, University Health Network, Toronto, ON M5G 2A2, Canada

**Keywords:** spasticity, spasticity modeling, wearable sensors, stretch reflex threshold, catch angle

## Abstract

Spasticity, a common symptom in patients with upper motor neuron lesions, reduces the ability of a person to freely move their limbs by generating unwanted reflexes. Spasticity can interfere with rehabilitation programs and cause pain, muscle atrophy and musculoskeletal deformities. Despite its prevalence, it is not commonly understood. Widely used clinical scores are neither accurate nor reliable for spasticity assessment and follow up of treatments. Advancement of wearable sensors, signal processing and robotic platforms have enabled new developments and modeling approaches to better quantify spasticity. In this paper, we review quantitative modeling techniques that have been used for evaluating spasticity. These models generate objective measures to assess spasticity and use different approaches, such as purely mechanical modeling, musculoskeletal and neurological modeling, and threshold control-based modeling. We compare their advantages and limitations and discuss the recommendations for future studies. Finally, we discuss the focus on treatment and rehabilitation and the need for further investigation in those directions.

## 1. Introduction

Upper motor neuron syndrome (UMNS) is a set of symptoms arising from damage to the descending motor pathways from the motor cortex to the spinal cord. UMNS can be a result of stroke, brain injury or spinal cord injury (SCI), or neurological disorders including cerebral palsy and multiple sclerosis (MS). Some potential symptoms seen in UMNS include muscle weakness, reduced muscle endurance, hypotonia (decreased muscle tone) or hypertonia (increased muscle tone). The damage to the neural pathway can also affect the motor control of an affected person, resulting in worsened speed or accuracy of movement. Spasticity is a common symptom of upper motor neuron lesions, which can be described by increased muscle tightness and stiffness, and a hyperexcitability of the reflexes that causes involuntary contraction of the muscles or jerky movements. Spasticity presents in varying degrees of severity, and it can interfere with the daily activities, movement or speech of an affected person, and can cause discomfort or pain [1]. Approximately 42% of stroke patients develop spasticity within six months of the onset of stroke [2], and spasticity affects about 65% of patients with MS [3], and about 70% of individuals living with SCI [4].

Despite the general understanding of spasticity, there is a lack of a commonly accepted definition, especially in neurology and biomechanics societies. In a review of 250 studies [5], 35% simply equate spasticity with increased muscle tone, and nearly the same number either fail to define it or use their own definitions of spasticity. However, over 30% of studies refer to Lance’s [6,7] definition of spasticity, i.e., “a velocity-dependent increase in tonic stretch reflexes with exaggerated tendon jerks, resulting from hyper-excitability of the stretch reflexes.” Indeed, increased muscle tone, its velocity-dependency and stretch reflex hyper-excitability are essential in characterizing spasticity. Rigidity is a symptom commonly seen in Parkinson’s disease, characterized by increased muscle resistance, which occurs through the range of motion [8] and neither depends on the velocity nor the acceleration of movement [1], which is similar yet fundamentally different from spasticity. Spasticity also needs to be distinguished from clinically defined flexor synergy as described by Twitchell [9] and Brunnstrom [10]. An example of flexor synergy in the clinical sense, which should not be mistaken with synergy in the context of motor control, is an abnormal coactivation of shoulder abductor muscles with elbow flexor muscles [11]. Other factors may contribute to increased muscle activity seen in spasticity, such as cutaneous or pain-related reflex mechanisms [12]. Therefore, it is important to differentiate spasticity from other symptoms and identify and follow a consistent definition.

There is also a lack of consensus about the mechanisms involved in spasticity [12]. For instance, what neurological and physiological factors are involved, and how much do they contribute to the condition? It is challenging to answer the above question as the lesions affect different pathways in a patient-specific way and the subsequent adaptation in the spinal network varies across patients. The contribution of the spinal excitatory and inhibitory mechanisms and supraspinal (both inhibitory and excitatory) pathways to spasticity are still not fully understood and require further investigation.

Assessing spasticity is important for patient follow up, especially to evaluate the effectiveness of treatments by medication or rehabilitation [13,14], and to improve our understanding of the underlying factors of spasticity. In this paper, we present a review of the current state of research and application in assessing spasticity. Instead of a systematic review of the overall research, we focus on identifying different approaches for quantitative modeling of spasticity. Representative papers were selected based on modeling approaches to demonstrate the differences between the methodologies. We describe the subjective measures currently used in the clinical field in Section 2. Section 3 describes the different objective approaches and the key measures used. In Section 4, we briefly discuss the approaches, their strengths and limitations, challenges, and future directions for spasticity research.

## 2. Subjective Clinical Measures

Qualitative measures are widely used in the clinical setting to assess spasticity, most notably the Modified Ashworth Scale (MAS) [15,16] (see Table 1), which tends to be subjective, relies heavily on the examiner’s experience, and is inaccurate, especially for the lower limbs [17]. Even for upper limbs, there is inconsistency in the inter-rater and intra-rater reliability. While some studies reported good inter- and intra-rater reliability [16,18], mostly for upper limb muscles, others reported poor reliability between raters [19]. Reporting the reliability based on only two raters, as has been done in those studies, is questionable. Pandyan et al. [17] in their review suggest that both the Ashworth Scale (AS) and MAS are only good as ordinal and nominal level measures of resistance to passive movement, respectively, not measures of spasticity itself.

Such clinical scores are also unable to differentiate the previously discussed overlapping symptoms seen in UMNS. The scores are also blind to the factors that cause spasticity, therefore they could not contribute to understanding the underlying phenomena.

The Tardieu Scale is another clinical measure of muscle spasticity that can better account for the velocity-dependent characteristic of spasticity, by assessing the passive muscle response at slow and fast speeds. The Tardieu Scale and its modified version have been recently preferred over AS and MAS [20], as they better follow Lance’s definition and are more sensitive to the changes in spasticity [21,22]. However, there is a lack of investigations of the Tardieu Scale reliability and quality as a measure of spasticity [23].

The lack of consistency and reliability of subjective measures suggest a need for an objective measure based on a quantitative approach to accurately estimate spasticity. Such objective measures could be better suited for assessment and monitoring the subsequent treatment and rehabilitation of the symptom.

## 3. Objective Approaches

To address the shortcomings of existing clinical scores, objective measures of spasticity have been investigated in recent decades. In this section, we discuss the objective approaches that have been employed to characterize spasticity, where different sensor-based quantitative measurements have been used (detailed in Section 3.1) along with different modeling techniques (described in Section 3.2) to produce outcome measures that indicate the severity of spasticity.

### 3.1. Sensors and Measurements

#### 3.1.1. Electromyography

Electromyography (EMG) measures the electrical current generated in the muscles during contraction, and the signal can represent the activity of a given muscle [24]. The EMG signal is the product of a complicated process involving the nervous system and physiological properties of the muscles. The signal becomes noisy due to traveling through different tissues. Surface EMG (sEMG) uses electrodes on the skin to collect these signals, which makes it particularly prone to muscle crosstalk. Improper placement of sEMG electrodes can cause significant variations in the signal amplitude and spectral characteristics. These variations between recordings could be mistakenly attributed to the effects of a treatment or rehabilitation method [25]. Electrodes that are not aligned with the muscle fibers can also result in sEMG signals with distorted amplitude or frequencies. Staudenmann et al. [26] found that properly aligned bipolar electrodes result in the lowest root mean square difference between measured muscle forces and estimate muscle forces using the sEMG recordings. Despite the potential complications, the benefit of sEMG is the ease of use and reduced discomfort when compared to invasive techniques such as intramuscular EMG involving a needle.

Repeatability of sEMG recordings and analysis is necessary for any objective approach. Steele et al. [27] demonstrated the high repeatability of sEMG recordings and analysis of muscle synergies between clinical visits up to six weeks apart, suggesting that any change in the results of signal analysis reflects a real change in the muscular activity. Accurate detection of the onset of muscle activity is important in some approaches to quantitative modeling of spasticity, as described in Section 3.2. This emphasizes the importance of EMG-based event detection algorithms.

Staude and Wolf [28] investigated three representative methods for “event” detection in EMG signals: the traditional finite moving average (FMA), two-threshold (TT) criterion, approximated generalized likelihood ratio (AGLR), as well as the cumulative sum (CUSUM) type model (with known parameters) as a comparative reference for optimal performance. The FMA algorithm uses a sliding window technique, comparing the (weighted) mean amplitude of the data to a threshold value. The TT algorithm is essentially based on the sum of two subsequent squared observations, which must pass two threshold comparisons. The AGLR algorithm gains substantial estimation performance with prior knowledge about the dynamic variance profile associated with a muscle activation accounted for at the expense of more samples required [28]. The methods were compared to a model-based (dynamic process) algorithm for better detection performance relative to the traditional methods. They compared the estimated onsets of muscle activity to the true onsets; however, their definition of the “true onsets” is unclear.

#### 3.1.2. Kinematics, Force and Torque

Most investigations that quantitatively assessed spasticity used a mechanized apparatus, e.g., [29,30,31,32,33], which supports the limb during the experiments. Such an apparatus allows manual or motorized movement of the limb while recording kinematic and torque data with the integrated sensors. This approach is functional and valid for preliminary research with high reliability, but the restrictive nature of such setups would not represent all aspects of real, natural movement in daily life. More importantly, the utilized devices are typically bulky, especially if they involve robotic components [32,33,34], which is not feasible for widespread application in the clinical setting.

Recent advancements of wearable sensors and technologies allow for ubiquitously accurate monitoring of our movement, activities and physical health [35,36,37,38,39,40]. In recent studies of spasticity, few have used a portable system in their experiments. Some of the existing portable systems utilize a flexible electrogoniometer (based on strain gauge mechanism) [41,42,43,44], which is a simple method for measuring the joint angles. However, the resulting measurements would not be robust to the sensor placement; for instance, if the sensor is not perfectly aligned with the frame of motion. Electrogoniometer measurements could also result in inaccurate estimation of joint angle when the axis of rotation is changing [45,46]. Additionally, measurement of joint angle with electrogoniometers relies on accurately identifying the center of rotation, e.g., of the knee joint [47], which changes with motion [48] and would be difficult to manually identify and track.

Inertial measurement units (IMU) were used in recent studies on spasticity [49,50,51]. IMU calibration procedures were developed [51,52] in order to correct for imperfect placement and orientation and to produce signals that accurately represent the real motion of the limbs under study. Estimation of joint axis and angle estimation using IMU measurements has been shown to be accurate and valid when compared to camera-based motion capture systems [53,54], or compared to magnetic tracking systems [55]. IMU-based measurement of human kinematics has also been demonstrated with high repeatability and validity, for instance in gait analysis [56,57], and when fused with other sensors [58], and in 3D joint angle estimation [55]. Even using a single IMU has been shown to result in accurate motion analysis in studies evaluating rehabilitation exercise performance [59,60]. As wearable sensors, IMUs are more convenient and practical for use in a clinical setting than bulkier or stationary alternatives such as the camera-based motion capture systems. IMU-based joint kinematics estimation is therefore beneficial for spasticity evaluation, due to its ease of use, reliability, and repeatability of the measurements. Wireless sEMG and IMU sensors have been combined to assess rehabilitation activities such as reaching, flexing movements and other exercises [61,62], where high intra- and inter-subject reliability were demonstrated for the measurements [63]. IMU and sEMG sensors have also been used for load estimation in the industrial setting and showed potential in estimation of the biomechanical overload risks for manufacturing workers [64].

Many of the studies discussed in this review included in their analysis the resistive force (in many cases represented with torque) generated by the spastic muscles being investigated. Detailed in Section 3.2, some studies aimed to model and estimate the reflexive force and the EMG activity, as they reflect the magnitude of the spastic response to muscle stretch. The force or torque was also related to other outcome measures to characterize spasticity. As mentioned previously, the joint torque was often measured by a torque sensor in the experimental apparatus, otherwise the reactive force was measured by a multi-degree-of-freedom force sensor, or torque estimated by other sensors such as a dynamometer [34] or differential pressure sensor [65].

### 3.2. Quantitative Models

This section describes three quantitative modeling approaches of spasticity and different examples of each approach. The reviewed studies grouped based on their modeling approaches can be found in Table 2, Table 3 and Table 4, along with details on used sensors, methods, and computed measures.

#### 3.2.1. Mechanical Models

**Figure 1:**
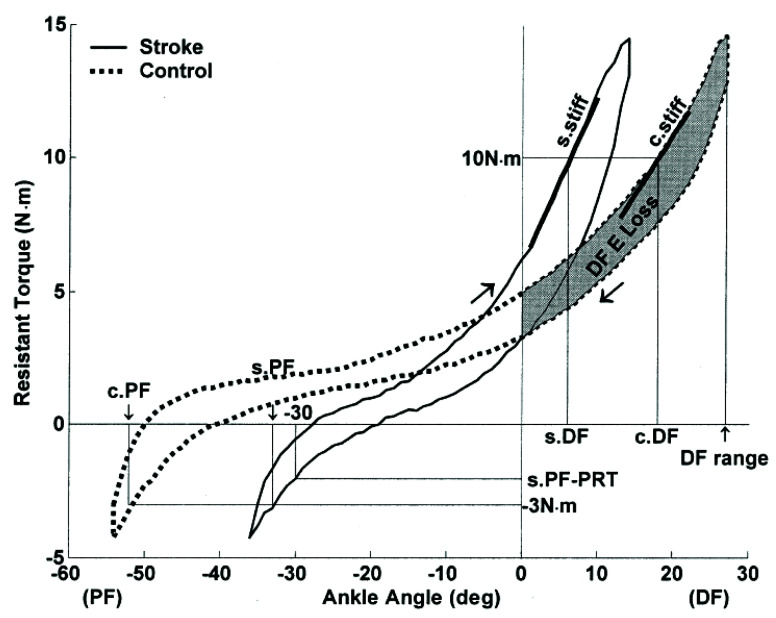
Representative torque-angle curves (hysteresis loops) from the experiments of Chung et al. [30]. The limit of dorsiflexion range of motion (ROM) was designated as the point of 10 Nm of resistive torque in both stroke and control subjects. The quasi-stiffnesses are the s.stiff and c.stiff slope values, respectively, for the stroke and control subjects.

Several studies approached modeling spasticity from a purely mechanical perspective [30,31,65,66,67]. Chung et al. [30] measured the resistive joint torque and angular position of the hemiplegic spastic ankle during passive dorsi- and plantar flexion motion. The slope of the torque-angle curve (see Figure 1) at the dorsiflexion ROM limit represented the quasi-stiffness of the ankle joint. Additionally, the area inside the curve across the dorsiflexed ROM represented the energy loss during dorsiflexion, which was then normalized by the ROM limit. Higher stiffness and energy loss indicate higher resistive forces during the joint motion, suggesting severe spasticity. The resistive torque at the nominal limits of plantar flexion and dorsiflexion, as well as the ROM, were also considered as outcome measures, where a smaller ROM and higher torque would suggest more severe spasticity. The participating stroke subjects showed significantly higher resistive torque, stiffness, and energy loss, as well as lower ROM when compared to healthy control subjects. These passive biomechanical properties had moderate to low correlation with the MAS scores (Kendall τ = 0.294, 0.297, 0.230 for torque, quasi stiffness, and energy loss, respectively; *p* < 0.05) [30], thus could provide informative measures of the spasticity in the muscles acting on the ankle joint.

Spasticity is typically characterized by the velocity-dependent increase in muscle tone, and the “catch”—the joint angle where the increased tone suddenly appears during fast passive stretching of the muscle [79]. To model this changing of muscle tone, Park et al. [66] divided the stretching motion of the elbow into three phases: pre-catch, catch, and post-catch. For the pre-catch phase, the passive elbow resistance was modeled as a linear mass-spring-damper system:

(1) $$\tau_{pre}=m\ddot{\theta }+b\dot{\theta }+k\theta$$

where *m* is the inertial mass of the hand and forearm, and *b* and *k* are the damping and stiffness, respectively. The catch angle can be represented as:

(2) $$\theta_{catch}=\theta_{i}+\frac{L}{{\dot{\theta }}_{pre}}$$

where *L* is the catch angle constant, $\theta_{i}$ is the angle at the beginning of the stretching motion, and ${\dot{\theta }}_{pre}$ is the average speed during the pre-catch phase. During the catch phase the elbow resistance was modeled as:

(3) $$\tau_{catch}=h{\dot{\theta }}_{c\_start}\delta \left(t\right)+\tau_{pre\_end}\;\;\;\;\;\;\delta \left(t\right)=\{\begin{array}{c} 1\;\;\;\;\;\;\;\;\;\;\;\;\;\;\;\;\;\;\;\;if\;t-t_{c\_start}<\;\Delta t_{c}\\ q\;\;\left(q<1\right)\;\;\;if\;t-t_{c\_start}\;\geq \;\Delta t_{c} \end{array}$$

where *h* is the catch torque constant, ${\dot{\theta }}_{c\_start}$ is the stretching speed at the beginning of the catch phase, $\tau_{pre\_end}$ the torque at the end of the pre-catch phase, *q* the residual torque constant, $t_{c\_start}$ the time when catch begins, and $\Delta t_{c}$ the duration of peak torque. Finally, the elbow resistance during the post-catch phase was represented as a position-dependent torque:

(4) $$\tau_{post}=k_{post}\left(\theta -\theta_{post\_start}\right)+m\ddot{\theta }+b\dot{\theta }$$

where $k_{post}$ is the stiffness and $\theta_{post\_start}$ is the initial joint angle of the post-catch phase. The model was based on analyzing the kinematics and force measurements during passive elbow stretching with four cerebral palsy (CP) participants. The MAS scores of the subjects were also assessed, and the complete model was used to simulate each subject’s spasticity in a haptic device consisting of a robotic arm, motor, and controller. The clinicians then performed the MAS assessment on the haptic device, which simulated the other subjects that they had not assessed previously, to validate the results of the modeling.

#### 3.2.2. Musculoskeletal and Neural Dynamics Models

Previous reviews on the objective characterization of spasticity showed the importance of differentiating the mechanical (musculotendon) and neural components of spasticity, especially for monitoring the effects of treatment or rehabilitation [80,81]. Obtaining those components requires the inclusion of both biomechanical and electrophysiological signals in the assessment of spasticity.

To model the neural and physical components of spasticity, several studies have designed theoretical controllers that include the musculoskeletal geometry, musculotendon dynamics, muscle spindle, motor neuron pool and subsequent muscle activations. The theoretical controllers receive the measured kinematics as inputs to estimate the force [68] or torque [34,69,70,71] generated by the muscles (due to reflex) for a given passive movement. The controller parameters consist of neural and non-neural parameters (e.g., muscle spindle firing rate, passive viscoelasticity, etc.) and are optimized to fit to the measured data. The estimated force or torque is generally represented as a sum of the effects of inertial, gravitational, and active muscle forces [69]:

(5) $$\tau_{T}=\tau_{I}+\tau_{G}+\tau_{M}$$

where $\tau_{T}$ is the measured torque, $\tau_{I}$ represents the torque from the moment of inertias, $\tau_{G}$ is the torque generated by gravity, and $\tau_{M}$ is the muscle torque consisting of a passive and active element, as in the following equation [69]:

(6) $$\tau_{M}=\tau_{passive}+\tau_{active}$$

The passive torque is characterizable beforehand by a slow, passive movement (e.g., joint angle speed of 15 deg/s), which minimizes muscle activation, leaving only the passive parameters to be identified by fitting the measured torque-angle curve [69]:

(7) $$\tau_{passive}=r\left(\theta \right)\left(k_{E1}e^{kE2\Delta L}+B\dot{L}+F_{0}\right)$$

where r(θ) is the moment arm about the joint, L is the muscle length, $k_{E1}$ the coefficient of the elastic exponential curve, $k_{E2}$ the rate of change of the curve slope, B the viscosity coefficient, and $F_{0}$ the elastic curve shape parameter. The active torque generated by the muscle was calculated based on the Hill-type muscle model, such as in [69]:

(8) $$\tau_{active}=r\left(\theta \right)\Delta a\left(t\right)f_{v}\left(\dot{L}\right)f_{l}\left(L\right)$$

where $f_{v}\left(\dot{L}\right)$ the relation between moment and rate of change of muscle length, $f_{l}\left(L\right)$ the relation between moment and muscle length, and a(t) is the muscle activation function, which includes the muscle spindle and motor neuron pool models.

**Figure 2:**
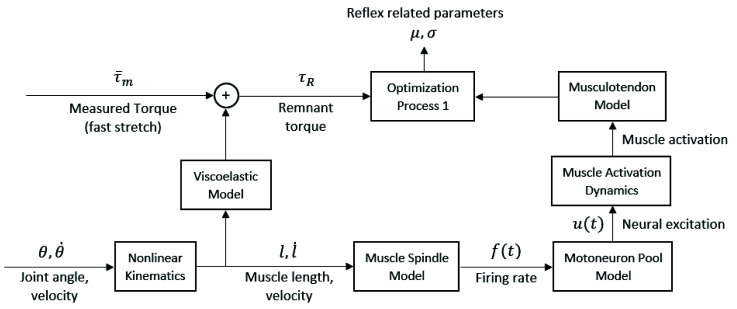
Example of system identification algorithm used by Shin et al. [69] for parameters characterizing the spastic reflexes, using muscle spindle, motor neuron pool, muscle activation dynamics, and musculoskeletal models to estimate the activate muscle torque generated by the spastic muscle during reflex.

**Figure 3:**
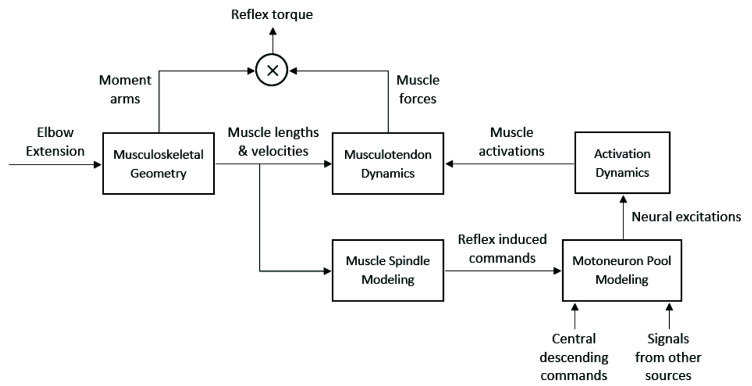
Another similar model used by Koo and Mak [34] that combines the moment arms of all the muscles that affect the joint movement being investigated with their active forces to estimate the resulting reflex torque.

Figure 2 and Figure 3 show examples of a theoretical controller used to indirectly estimate active torque generated by spastic reflex, allowing for identification of parameters related to the reflex. These models are used to estimate the measured experimental torque at the joint, and the optimized biomechanical and neural parameters of the controller are the outputs of this type of quantitative modeling which can characterize the level of spasticity. The models are complex and while they can be used to simulate spastic behavior, they may be less applicable in clinical evaluations.

#### 3.2.3. Threshold Control Models

Several spasticity models have been developed based on muscle reflex models and the stretch reflex threshold (SRT). One hypothesis of how the central nervous system (CNS) controls human movement is threshold position control [82], or in a more general form, the Equilibrium Point (EP) hypothesis [83]. The EP hypothesis suggests the CNS changes the relationship between length and force in muscles to reach a new position and force equilibrium where opposing muscle forces are balanced, resulting in movement or a static posture. Specifically, it assumes the CNS controls a motor action, whether single-joint or multi-joint, by modulating the thresholds or EPs, which results in transitioning between states along a planned trajectory. Spasticity can be defined as an involuntary, velocity-dependent increase in tonic stretch reflexes, or reduction in the threshold of muscle stretch at which the tonic reflex begins and muscle force increases as a function of length. Since spasticity distorts the tonic reflex thresholds, it can adversely affect the motion control, which can be described by the EP hypothesis.

**Figure 4:**
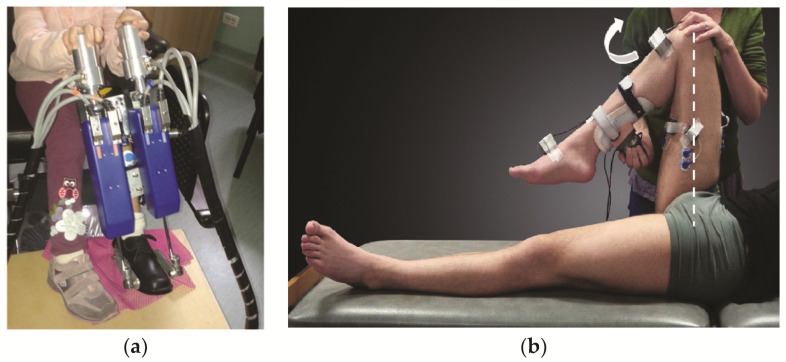
(**a**) Example of a motorized setup for stretching the ankle dorsi- and plantar flexors [32] and (**b**) a manual setup for extending the knee joint and stretching the flexor muscles [49].

Levin and Feldman [74] used sEMG recordings to detect the onset of the elbow flexor muscle activations as a result of spastic hyperexcitability during passive extension at different speeds. In their experiment, a motorized apparatus was used to hold and passively move a participant’s arm, while recording the kinematic data. Figure 4 shows an example of a motorized setup and an example of a manual setup for investigations of the lower extremities.

**Figure 5:**
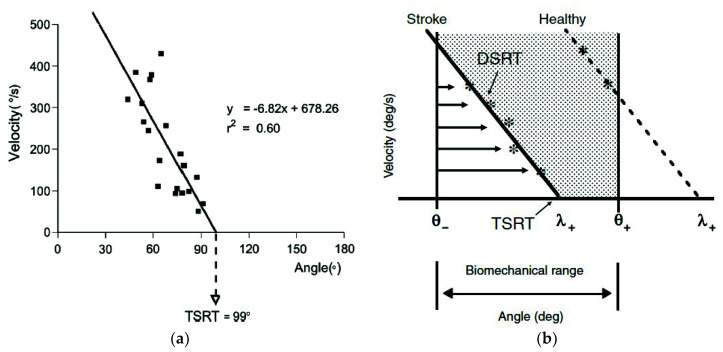
(**a**) Example of tonic stretch reflex threshold (TSRT) estimation by 20 dynamic stretch reflex threshold (DSRT) points found by stretching the elbow flexor muscle biceps brachii at different velocities; (**b**) example of a threshold model for a post-stroke subject versus healthy person, where the TSRT lies outside the biomechanical range of the joint [43].

The joint angular velocity and the joint angle at the onset of spasm was used to define the dynamic stretch reflex threshold (DSRT). Repeating passive elbow extension multiple times at different speeds allowed for data-driven modeling, building a linear regression model on the combined data for a given motion and associated muscle(s). The intercept of this linear model (Equation (9)) with zero velocity represents the tonic stretch reflex threshold (TSRT) (see Figure 5a). Several other investigations used this fundamental approach to evaluate spasticity [1,32,41,42,43,51,73,76], based on the following equation [74]:

(9) DSRT=TSRT−μ×Velocity

where μ represents the sensitivity of the dynamic stretch reflex threshold to velocity, and a higher μ means greater spasm sensitivity to velocity.

For an individual with spasticity, the TSRT of an affected muscle is shifted within the biomechanical range of motion of a joint, even at a relax state, preventing movement throughout the full range. In contrast, the TSRT for a healthy individual or unaffected muscle would lie outside the ROM. This is supported by their dynamic stretch reflexes only appearing at higher potential velocities (see Figure 5b), in a case such as a knee tendon tap, which evokes a similar response to a very high stretch velocity of the quadriceps muscles, in excess of 300 deg/s [73,84]. Therefore, as the quantitative outputs of the model, a lower TSRT value and higher μ value would suggest more severe spasticity for a specific muscle. Combining the models for the muscles acting on a particular joint can provide a map of the spastic joint space [51].

The mentioned studies, including the purely mechanical approaches and musculoskeletal and neural dynamics models, investigated spasticity through passive-movement experiments. However, in daily life situations and outside of the lab setting, spasticity could also be triggered due to active movement. Thus, it is important to extend the scope of experiments to include active movements to characterize and assess spasticity in a more comprehensive capacity.

**Figure 6:**
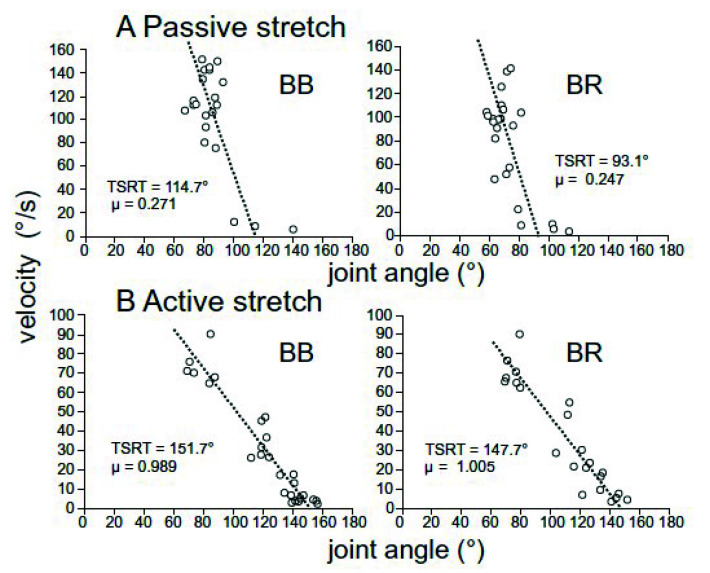
A representative subject in [84] where in the active stretching of elbow flexors—biceps brachii (BB) and brachioradialis (BR)—the TSRTs were found to occur at greater joint angle or higher stretch. In contrast, the sensitivity to velocity was found to be increased in both muscles, when compared to passive motion.

According to the threshold control theory, some believe that spasticity can be described as an impaired ability to regulate the tonic stretch reflex thresholds, and recent studies have begun to investigate this concept. Turpin et al. [84] tested both passive flexion and extension of the elbow joint by an experimenter, and with active, volitional elbow motion in identical conditions. Passive and active movements were performed with the same range of motion specific to each participant at a variety of joint angular velocities. Obtained TSRTs were at greater angular displacements, corresponding to more stretched muscles, in the active stretching compared to the passive stretching (by 10–40 deg), suggesting an increase in non-spastic ROM. Conversely, the slopes of the regressions (parameter *µ*) were increased by 1.5 to 4.0-fold, showing a higher sensitivity to velocity during volitional control. These findings suggest that during volitional motion an affected individual could stretch the muscle/extend the joint further than the during passive motion, particularly at slower speeds. However, at greater velocities the DSRTs estimated from active and passive motions are in a similar range. Figure 6 shows a representative subject from that study.

In an earlier study on implicit learning and generalization for stretch reflexes in healthy subjects, Turpin et al. [75] found the amplitude of the stretch reflex decreases and remains attenuated by 5–12 repeated stretches, and does not increase even after 5 min of rest. This observation can be explained by the anticipation of the stretching which can result in the pre-modulation of spatial thresholds that can suppress the muscle resistance to stretch. This pre-tuning of stretch reflex is similar to the clasp-knife phenomenon [85] seen in individuals with Parkinson’s disease and stroke survivors who have rigid or spastic muscles [86,87]. 

## 4. Discussions

### 4.1. Comparing the Modeling Approaches and Future Directions

Subjective measures, most commonly the AS and MAS, as well as other clinical scores, are currently used to assess spasticity in clinical practice. These scores are easy to obtain and do not require any equipment and sensors, unlike the objective approaches. However, the issue remains of their questionable reliability, weak correlation with muscle activity measurements of the reflexes [73,88,89], and inability to reflect the complex mechanisms of the spastic reflexes. Despite these shortcomings, subjective measures should not be totally abandoned until a reliable, objective measure is found and established, but they need to be supplemented with current quantitative approaches.

The mechanical modeling approaches represent spasticity at the joint level, usually in joint torque-kinematic space, whether by identifying biomechanical properties that differ between a healthy individual and an individual with spasticity (e.g., change in joint mechanical impedance) or representing the spastic behavior by a simulation model. The outcomes have been shown to moderately correlate with clinical scores such as the MAS [30], demonstrating the potential of this type of approach, which is also simpler and easier to use in a clinical setting than more complex modeling approaches. However, as previously discussed, the assessment of spasticity for follow up and treatment is better accomplished by differentiating biomechanical and neural components of spasticity, using both mechanical variables and electrophysiological signals [80,81].

The resulting biomechanical and neural parameters of the musculoskeletal and neural dynamics models allow for characterizing spasticity at the muscle level. The obtained measures may allow understanding of some aspects of the neurophysiology of spasticity, and could potentially be applied to the development of treatments. For example, Shin et al. [69] arrived at optimized parameters *μ* which represents the muscle spindle firing rate at 50% motor neuron recruitment, and *σ* as the standard deviation of the Gaussian cumulative distribution that represents the function of the alpha motor neuron pool. A lower *μ* means a lower minimum spindle firing rate which indicates hyper-reflexia in the muscle [90]. The higher reflexive torque (increased muscle tone) found with lower *μ* and *σ* values in their experiments shows a possible relationship between those parameters and spasticity. Koo and Mak [34] showed similar results by looking at *μ_0_*, the minimum spindle firing rate for just 0.5% neural excitation, and *G_L_* as the muscle spindle static gain. These parameters were posited to be more physiologically meaningful in relation to spasticity. Using sensitivity analysis, *μ_0_* and *G_L_* were determined as the key parameters when predicting reflex torque. Koo and Mak suggested that drug or treatment development could be focused on effectively regulating those specific parameters. Clinical scores have been used besides this modeling approach to assess the subjects’ spasticity [70], and it was found that stiffness, viscosity, and reflex torque are positively correlated with AS scores. However, the authors did not include neural parameters in their torque estimation model, and instead used measured EMG to estimate the neuromuscular activity due to stretch reflex. Recent advancements in joint mechanical impedance estimation during active movements [91,92,93,94] would allow further investigations on how spasticity affects the modulation of joint impedance, particularly joint stiffness and viscosity, during volitional movement and walking.

From a research-oriented point of view, this type of investigation can provide meaningful details about spasticity. However, they are not likely to be clinically applicable, as also mentioned in other reviews of the literature [95], due to the complexity and time required for setup preparation and data processing. An easy to use objective assessment method that can still benefit from high level neural and mechanical modeling could provide a more suitable solution for spasticity assessment in the clinics. This high-level approach could be based on the threshold control-based models [1,32,41,42,43,51,73,74,76,84], which explicitly reflect the velocity-dependence of spasticity. These models have also been shown to be moderately correlated with clinical scores, agreeing with the current practice and are generally simpler than the methods that use parameter-based estimations of spastic responses. While these models are usually acquired with robotic setups which can be complex and not available in every clinic, several studies showed the potential of using wearable sensors and inexpensive hand-held instruments to obtain such models accurately [49,50,51].

Previous studies have found that the spastic reflex is affected by the initial stretch level at the beginning of a stretching motion, given the same stretching velocity [29,96]. Kamper et al. [29] found that with longer initial lengths of the elbow flexor muscles, the reflex threshold and stiffness were significantly reduced and increased, respectively, indicating a negative relationship between the initial muscle length and the spastic reflex. The approaches discussed in Section 3 do not account for this observation. Future studies should incorporate varying initial stretch positions in addition to varying stretch speeds in their investigations. 

More recent studies have found that the firing of muscle spindles is not necessarily unique in relation to muscle length and stretch velocity but may be more directly related to muscle force. Blum et al. [97] demonstrated that the instantaneous firing rates (IFRs) of muscle spindle primary afferents are significantly better predicted by force-related variables than muscle length-related variables, especially at higher stretch velocities. Falisse et al. [98] also found that estimating muscle activity (using EMG) during spastic reflexes in passive motion, as well as gait in children with CP, was better accomplished using measured force (applied by the examiner in passive motion and ground reaction forces in gait) than models that estimated using kinematics variables. For instance, the activity of the hamstrings was predicted significantly better in both cases by force than velocity or acceleration (R^2^ = 0.73 ± 0.10, 0.46 ± 0.15 and 0.47 ± 0.15, respectively). These results suggest a need for incorporating reflex generated muscle force or torque into the modeling of spasticity beyond that of estimating the measured profiles using other variables such as joint kinematics. Future investigations should aim to consider the relationship between muscle force and the spastic reflex in characterizing and assessing spasticity.

### 4.2. Effect of Spasticity Modeling on Follow-Ups and Treatment

As discussed previously, reliable and accurate assessment of spasticity by objective measures could lead to better follow-ups and treatment. Previous studies of treatment of spasticity have been limited by solely using clinical scores to evaluate the effects of the treatments. Simpson et al. [99] used the AS to evaluate the efficacy of botulinum toxin type A (BTX-A)—a common treatment option—on the upper limb spasticity in post-stroke subjects. The experiment was randomized, double-blind and placebo-controlled, but the limitations of the AS calls into question the results that showed significant reductions in spasticity. In a recent study by Turna et al. [100] the effects of different injection techniques of BTX-A were investigated for treating ankle plantar flexor spasticity. To compare those techniques the effects of the treatment were evaluated with subjective scores including the AS, Brunnstrom stages, and Barthel index score, which again limits the reliability of the results.

Some studies have initially explored the idea of investigating the effects of treatments and management of spasticity by objective measures. Chen et al. [65] compared the spasticity in the affected biceps-brachii muscle in ten chronic stroke patients, two weeks before and after BTX-A injection. Measured by a portable device, the elbow joint kinematics, reactive torque and muscle activity were analyzed to estimate the viscosity of the muscle and the DSRTs (as a percentage of the stretch cycle). They found a significant decrease in viscosity and a significant increase in DSRT after injection. The results indicated a reduction in spasticity, which agreed with their MAS assessments performed before and after the treatment. However, Pandyan et al. [15] identified reductions in spasticity in the elbow flexors of stroke patients, which were not detected by the MAS assessments. These results reinforce the idea that clinical scores offer an insufficient and unreliable evaluation of spasticity. A better measure of spasticity can be obtained by employing quantitative evaluations that provide objective, accurate measures of spasticity and offer models that can predict spastic behavior [65]. Investigations beyond this preliminary research could potentially reveal precise relationships between dosage and the effects, allowing for an optimally effective plan to be designed for each patient [15,65]. 

Several studies have investigated repetitive transcranial magnetic stimulation (rTMS) and functional electrical stimulation (FES) and their effect on spasticity. Several studies found that rTMS significantly reduces spasticity in the lower limbs, for instance, in SCI participants with the effects lasting up to a week as measured by the MAS [101], and in stroke patients [102] as measured by their own clinical scale. Franek et al. [103] found that FES improves spasticity in the hip adductors of subjects with SCI for a few days up to a few months, as evaluated by a subjective scale (scale of 1–6) and objective measures such as H reflex recruitment curves and the number and intensity of contractions, while Alfieri [104] found that not all their participants (varying cases with hemiplegia and SCI) benefitted from FES. Powell et al. [105] found that FES improves wrist extensors strength and ROM, though not specifically for spasticity as evaluated by AS, and it was unclear how long the effects lasted. A review of ten recent studies [106] found that spasticity was significantly reduced in quadriplegic and paraplegic patients by treating with FES-cycling exercise. However, the effects were primarily evaluated by MAS. Overall, there is limited evidence of the benefit of FES for spasticity, and in many cases the utilized subjective scores and their lack of reliability (particularly for lower limbs) could have contributed to the mixed results. Objective measures of spasticity, such as the DSRT, could better evaluate and potentially prove the usefulness of FSE and rTMS for alleviating spasticity in conjunction with other treatments or rehabilitation [107].

## 5. Conclusions

This paper reviewed different approaches used for modeling spasticity, with a focus on objective and sensor-based systems. Approaches that use purely mechanical modeling can provide some information on the biomechanical properties of spastic behavior but lack consideration for the neural factors of spasticity and electrophysiological activity. The musculoskeletal and neural dynamics models can provide insight into the detailed mechanisms of spasticity, such as the theoretical neural parameters involved in the spastic reflex but lack practicality and applicability in the clinical environment. The threshold control-based models can provide an easy-to-use objective method of assessment, especially with wearable sensors in the clinical setting. However, further investigations into the neural mechanisms involved in spasticity may prove beneficial for better understanding and assessing spasticity.

There is a need to develop a system that can provide an objective, accurate and reliable assessment of spasticity—especially in the lower limbs—to better evaluate the effects of treatment and rehabilitation options. Identifying an accurate and objective spasticity model for each patient allows for predicting the kinematic states that provoke spastic behavior. Such a model could inform rehabilitation programs and enable adapting the assisted movements provided by a physiotherapist or an assistive exoskeleton so that uninterrupted exercises may be achieved. Obtaining spasticity-free assisted exercises has the potential to remarkably improve the outcomes of physical rehabilitation.

## Figures and Tables

**Table 1:** Modified Ashworth Scale (MAS) [16].

Grade	Description
0	no increase in muscle tone.
1	slight increase in muscle tone, manifested by a catch and release or by minimal resistance at the end of the range of motion (ROM) when the affected part(s) in moved flexion or extension.
1+	slight increase in muscle tone, manifested by a catch, followed by minimal resistance throughout the remainder (less than half) of the ROM.
2	more marked increase in muscle tone through most of the ROM, but affected part(s) easily moved.
3	considerable increase in muscle tone, passive movement difficult.
4	affected part(s) rigid in flexion or extension.

**Table 2:** Reviewed mechanical approaches to modeling spasticity.

Authors	Target Population	Target Joints	Sensors	Method	Outcome Measures
Alibiglou et al. [31]	Post-stroke	Elbow and ankle	Non-wearable 6-axis force sensor, potentiometer, tachometer	Motor-driven motion; system identification model; goodness of fit evaluated by percent variance accounted for (%VAF)	Intrinsic stiffness, reflex stiffness; near-zero correlation with MAS
Chen et al. [65]	Post-stroke	Elbow	Wearable gyroscope, differential pressure sensor, sEMG sensors	Manually driven motion; phase-shifted torque-angle curve	Average viscosity (across multiple stretching speeds), muscle activity onset
Chung et al. [30]	Post-stroke	Ankle	Non-wearable 6-axis force sensor, unspecified kinematics sensors	Motor-driven motion; torque-angle curves	Resistance torque, quasi-stiffness, energy loss and ROM; low to moderately correlated with MAS
Park et al. [66]	CP (children)	Elbow	Unspecified kinematics and force sensors	Manually driven motion; model of torque during pre-, during, and post-catch phases	Replication of MAS level on simulated spastic elbow (haptic device); model accuracy evaluated by blinded assessors
Wu et al. [67]	Post-stroke	Elbow	Non-wearable potentiometer, torque sensor; wearable sEMG sensors	Manually driven motion; torque-angle curve, 4-D characterization of catch angle using torque, torque rate of change, angle and velocity; model accuracy evaluated by mean square error	ROM, stiffness, energy loss, catch angle; high correlations with MAS

**Table 3:** Reviewed musculoskeletal and neural dynamics approaches to modeling spasticity.

Authors	Target Population	Target Joints	Sensors	Method	Outcome Measures
Koo and Mak [34]	Post-stroke	Elbow	Non-wearable dynamometer and needle EMG electrode; wearable sEMG sensors	Motor-driven motion; parameter identification in torque estimation and sensitivity analysis; model goodness of fit evaluated by root mean square error (RMSE)	Minimum spindle firing rate for 0.5% neural excitation, muscle spindle static gain
Lindberg et al. [68]	Post-stroke	Wrist	Non-wearable stepper motor, unspecified force sensor; wearable sEMG sensors	Motor-driven motion (multiple speeds); force estimation to separate into components; re-test with ischemic nerve block	Neural component (NC) of force—model validated by NC reduces with ischemic nerve block and velocity dependence of NC; moderate correlation between NC and MAS, also integrated EMG
Shin et al. [69]	Post-stroke	Ankle	Non-wearable torque sensor, rotary encoder; wearable sEMG sensors	Manually driven motion; parameter identification in torque estimation; model goodness of fit evaluated by %VAF, normalized RSME, and *R*^2^	Muscle spindle firing rate for 50% motor neuron recruitment, standard deviation of alpha motor neuron pool function
de Vlugt et al. [70]	Post-stroke	Ankle	Non-wearable potentiometer, force transducer; wearable sEMG sensors	Motor-driven motion (multiple speeds); parameter identification in torque estimation; model goodness of fit evaluated by %VAF, performance by repeatability	Stiffness and viscosity parameters; stiffness moderately correlated with AS at low speed, reflex torque moderately correlated with AS at fast speeds
Wang et al. [71]	Post-stroke	Wrist	Non-wearable force transducer, high-precision stepper motor; wearable sEMG sensors	Motor-driven motion (slow and fast speed); parameter identification in torque estimation; model goodness of fit evaluated by %VAF and *R*^2^	Passive stiffness, muscle spindle firing rate for 50% motor neuron recruitment, motor neuron pool gain

**Table 4:** Reviewed threshold-control approaches to modeling spasticity.

Authors	Target Population	Target Joints	Sensors	Method	Outcome Measures
Arami et al. [51]	Incomplete SCI	Ankle	Wearable IMUs, 6-axis force sensors, wireless sEMG sensors	Manually driven motion at different knee angles; DSRT model for dorsi- and plantar flexor muscles; models goodness of fit evaluated by *R*^2^	Model *μ* and TSRT for each muscle; spastic joint space; joint torque moderate-high correlation with DSRT angle and velocities
Bar-On et al. [49]	CP (children)	Knee and ankle	Wearable IMUs, 6-axis force sensors, wireless sEMG sensors	Manually driven motion; DSRT model and torque-angle curve; model evaluated by repeatability	ROM, max velocity, average RMS-EMG, torque, and work
Blanchette et al. [42]	Post-stroke	Ankle	Wearable electrogoniometer, sEMG sensors	Manually driven motion; DSRT model for plantar flexors	Model *μ* and TSRT; interrater reliability for TSRTs
Calota et al. [43]	Post-stroke	Elbow	Wearable electrogoniometer, sEMG sensors	Manually driven motion; DSRT model of biceps brachii	TSRT; moderately good intra- and interrater reliability, no correlation with MAS
Germanotta et al. [32]	CP (children)	Ankle	Non-wearable mini-rail linear encoders, unspecified torque sensor; wearable wireless sEMG sensors	Motor-driven motion; DSRT models of dorsi- and plantar flexors; goodness of fit evaluated by *r* correlations	Model *μ* and TSRT; low to moderate correlations with MAS
He et al. [44]	MS	Knee	Wearable electrogoniometer	Pendulum test [72]; estimation of swing trajectory during pendulum test	DSRT, TSRT and stretch reflex gain
Jobin and Levin [73]	CP (children)	Elbow	Non-wearable angle and velocity transducers; wearable sEMG sensors	Motor-driven motion; DSRT models of elbow flexors and extensors	TSRT; high test-retest reliability by ICC, no correlation with CSI^2^
Kim et al. [41]	Post-stroke	Elbow	Wearable twin-axis electrogoniometer, sEMG sensors	Manually driven motion; DSRT models, K-means clustering of TSRT groups	Significant differences between K-means groups (3 levels), no significant differences between groups by MAS; very high correlation between K-means groups and TSRTs
Levin and Feldman [74]	Post-stroke	Elbow	Non-wearable precision digital resolver; wearable sEMG sensors	Motor-driven motion; DSRT models of elbow flexors and extensors	Model *μ* and TSRT; moderate correlations with MAS
Mullick et al. [1]	Post-stroke, Parkinson’s	Elbow	Non-wearable precision axial gauge; wearable sEMG sensors	Motor-driven motion ^1^; DSRT models of elbow flexors and extensors; goodness of fit evaluated by *R*^2^	Sensitivity of DSRT to velocity – high for post-stroke, near-zero for parkinsonian; zero correlation between *μ* and TSRT and CSI ^2^
Turpin et al. [75]	Post-stroke	Elbow	Non-wearable optical encoder; wearable sEMG sensors	Manually driven (passive) and active motion; DSRT models of flexors and extensors	Velocity sensitivity *μ* and TSRT increased in active stretch; change in TSRT between passive and active was moderate to highly correlated with CSI ^2^ and FMA ^3^
Zhang et al. [76]	Post-stroke, brain trauma, SCI	Elbow	Wearable IMUs and sEMG sensors	Manually driven motion; DSRT model of flexor muscle, reconstructed models of kinematic profiles; supervised single/multi-variable linear regression and support vector regression	Predicted evaluation scores (MAS) using TSRT, biomarkers from kinematics models, and combination of both; models estimation performance evaluated by mean square error

^1^ Velocity profile was bell-shaped (more natural), other motor-driven apparatus used ramp-shaped motion; ^2^ Composite Spasticity Index [77]; ^3^ Fugl-Meyer Arm Assessment [78].
